# Influencing factors related to stroke patients’ rehabilitation motivation: a scoping review

**DOI:** 10.3389/fneur.2025.1615905

**Published:** 2025-07-23

**Authors:** Xiaowen Fan, Yi Xia, Junrong Wu, Shulei Jia, Jiangyu Hu

**Affiliations:** School of Nursing, Jiangxi Medical College, Nanchang University, Nanchang, Jiangxi, China

**Keywords:** stroke, rehabilitation motivation, influencing factors, scoping review, rehabilitation

## Abstract

Mounting epidemiological evidence indicates a rising global prevalence of stroke among adults and older populations, often leading to severe functional impairments that compromise daily living. While rehabilitation exercise is recognized as a safe and effective strategy for functional recovery, inadequate rehabilitation motivation frequently undermines therapeutic efficacy. Exercise adherence plays a critical role in mitigating physical disability and mortality rates, yet current research lacks systematic insights into influencing of patient rehabilitation motivation. This scoping review synthesizes evidence from 23 studies across domestic and international databases to identify multidimensional factors influencing post-stroke rehabilitation motivation. Key influencing include intrinsic drivers (e.g., self-efficacy, depression), extrinsic supports (familial or social support, economic burden), disease-specific characteristics (severity, functional deficits), and intervention strategies (cognitive-behavioral therapy, gamified rehabilitation). Findings highlight the necessity of integrating personalized motivational assessments into clinical protocols and developing interdisciplinary interventions to address motivational barriers. These insights provide a foundation for optimizing rehabilitation frameworks and improving long-term patient outcomes.

## 1 Introduction

Stroke, a sudden rupture or obstruction of cerebrovascular structures caused by multiple factors (e.g., ischemic or hemorrhagic events), is a life-threatening condition that severely impacts human health and quality of life. It is characterized by a high incidence, mortality, recurrence, and disability (1, 2). According to statistics from the World Health Organization (WHO), approximately 15 million individuals worldwide experience a cerebrovascular accident annually, with around 5 million succumbing to severe stroke (3). Stroke patients are more likely to die if they do not receive treatment within the golden hour. In addition, even if they receive the right treatment at the right time, 70–80% of them will develop post-stroke disability (3, 4), such as loss of range of motion, abnormal posture, spasticity, memory deficits, spatial neglect, aphasia, and dyspraxia. While recovery is possible, most patients will remain with permanent limitations and impairments. All these factors seriously affect the future quality of life of patients (5). Rehabilitation therapy is currently the primary measure to reduce disability rates and serves as a safe intervention (6). Therefore, early, timely, and effective rehabilitation exercises are particularly important.

However, rehabilitation exercises are also influenced by several factors. For example, the cost of rehabilitation and the financial burden of not being able to carry out normal productive activities put pressure on the patient and family. Rehabilitation services are the main drivers of cost (6, 7). In addition, the unwillingness or inability of stroke patients to participate in exercise for physical and psychological reasons can affect their rehabilitation outcomes. Successful rehabilitation outcomes depend on the active participation of patients through their will and motivation.

Motivation is described as an essential factor in rehabilitation outcomes (7, 8). However, current rehabilitation literature indicates a lack of consensus regarding the conceptualization and underlying influence of motivation in clinical settings (9). Nevertheless, it is widely recognized by rehabilitation specialists that patient motivation serves as a critical prognostic factor influencing therapeutic outcomes. This psychological construct significantly contributes to treatment adherence and program continuity. Empirical evidence demonstrates that rehabilitation motivation functions as a primary catalyst for therapeutic engagement, with heightened motivational states correlating with proactive patient behaviors (10). Specifically, patients exhibiting strong rehabilitation motivation demonstrate increased initiative in acquiring exercise-related knowledge, enhanced adherence consciousness, and ultimately improved functional recovery rates with reduced disability incidence.

A comprehensive understanding of rehabilitation motivation mechanisms constitutes a critical precursor to optimizing therapeutic outcomes in recovery processes. Contemporary research has demonstrated significant scientific interest in identifying determinants affecting stroke rehabilitation efficacy, with extensive investigations conducted globally. While comparative analyses indicate substantial disparity in research attention between general stroke rehabilitation factors and post-stroke motivational drivers, an emerging evidence base nevertheless confirms the significance of motivation-related variables in neurological recovery procedure. To date, various studies have been conducted, including cross-sectional and longitudinal studies, qualitative and quantitative research, and a few reviews and meta-analyses (11–13).

Scoping reviews have become an increasingly prevalent method for informing decision-making and research by identifying and examining the literature on a specific topic or issue. These reviews incorporate evidence from various research methodologies and may also include information from non-research sources, such as policy documents. Consequently, scoping reviews offer a comprehensive overview that addresses broader research questions compared to the traditionally more focused systematic reviews of effectiveness or qualitative evidence (14).

Therefore, this paper will comprehensively organize and analyze the current factors influencing the motivation for rehabilitation for stroke, to provide reference information for improving the patients’ adherence to participate in rehabilitation, improving the clinical work of healthcare personnel, and improving the effectiveness of rehabilitation implementation, so as to improve the rehabilitation rate and reduce the disability rate after stroke.

## 2 Methods and analysis

To understand the factors influencing rehabilitation motivation in stroke patients and the strength of their correlation, a scope review was conducted to synthesize and analyze the research trends of related papers at home and abroad, and then a comprehensive analysis was conducted to determine the strength of the correlation between the influencing factors and the motivation to rehabilitate.

The following were the scoping review procedures.

### 2.1 Stage 1. Identifying the research question

The operational definition of research questions constitutes the methodological cornerstone of systematic review implementation, given that the establishment of eligibility criteria fundamentally derives from this conceptual framework. This investigation specifically addresses the following research question: What are the factors influencing motivation for rehabilitation in stroke patients and what is their distributional characteristics?

### 2.2 Stage 2. Identifying relevant studies

This study examines articles published from January 2015 to December 2024, focusing on academic papers and theses concerning the motivation for rehabilitation among stroke patients.

Building search expressions based on MeSH Database, the search terms comprised the MeSH and free text.

1. Stroke-related terms (MeSH + free-text):

("Stroke"[MeSH] OR "Cerebrovascular Accident"[tiab] OR "CVA"[tiab] OR "cerebrovascular apoplexy"[tiab] OR "brain vascular accident*"[tiab] OR "acute stroke"[tiab])

1. Rehabilitation motivation terms (MeSH + free-text):

AND ("Motivation"[MeSH] OR "Patient Compliance"[MeSH] OR "Treatment Adherence and Compliance"[MeSH] OR "rehabilitation motivation"[tiab] OR "rehabilitation adherence"[tiab] OR motivat*[tiab] OR incentive*[tiab] OR disincentive*[tiab] OR expect*[tiab])

1. Exclusion criteria:

NOT ("Habilitation"[MeSH] OR "Congenital Disorders"[MeSH]).

### 2.3 Stage 3. Study selection

**Figure 1:**
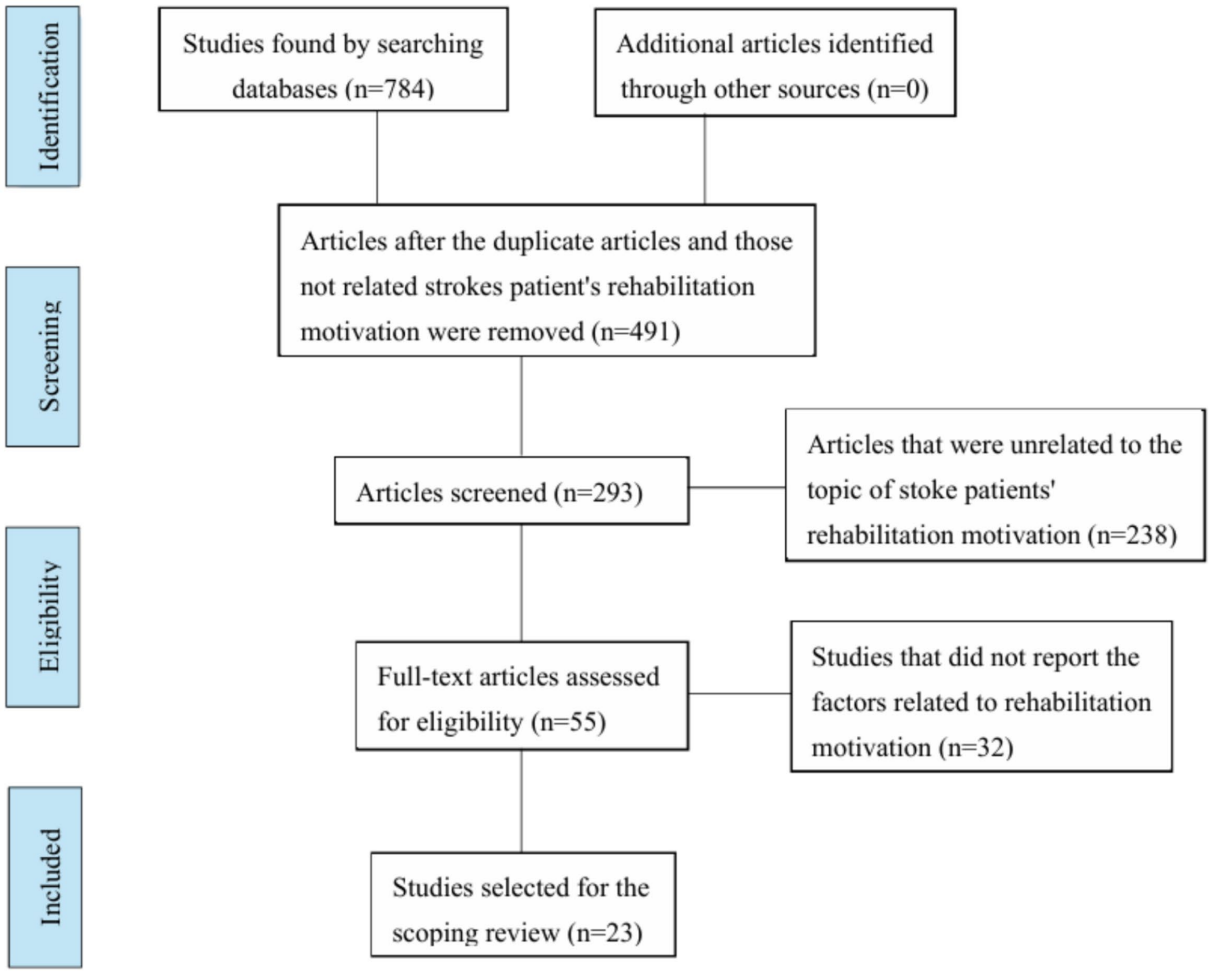
PRISMA flow diagram.

The study selection process was rigorously executed by two independent investigators (A and B) in accordance with predefined scoping review protocols. Following deduplication procedures, a four-phase screening framework was implemented: (1) primary literature identification, (2) initial title-abstract screening, (3) full-text eligibility assessment, and (4) dual independent appraisal with documentation verification. Any disagreement regarding the study selection was resolved through discussion. Following rigorous application of the eligibility criteria, 23 articles were ultimately included for analysis. The selection workflow was structured in compliance with the PRISMA Extension for Scoping Reviews (PRISMA-ScR) guidelines, as illustrated in Figure 1 (15). Our systematic search protocol targeted publications from January 2015 to December 2024, executed during February 2025 across nine multidisciplinary databases. Initial retrieval yielded 784 records distributed as follows: Web of Science (n = 96), PubMed (n = 77), Google Scholar (n = 35), Springer LINK (n = 127), Wiley Online Library (n = 10), Elsevier ScienceDirect (n = 266), CNKI (n = 44), CQVIP (n = 40), and Wanfang Data (n = 89).

Primary screening excluded 491 records failing to meet content relevance criteria. Subsequent abstract review eliminated 238 non-conforming articles due to either (a) irrelevance to stroke rehabilitation motivation mechanisms, or (b) absence of reported motivational determinants. This resulted in 55 articles progressing to full-text evaluation. Following deduplication procedures identifying 32 redundant publications, the final analytical corpus comprised 23 methodologically qualified studies.

### 2.4 Stage 4. Charting the data

Following scoping review methodology, we systematically organized key research elements into a comparative matrix to document study characteristics. As per Armstrong et al.’s methodological framework, this analytical tool categorizes scholarly works through nine critical dimensions: authorship, temporal context (publication year), research location, intervention typology, participant demographics, research objectives, methodological approaches, result interpretation, and conclusions (16). This structured approach enables systematic documentation of thematic convergences and divergences across selected studies. Consistent with this, data charting in this study included the first author’s name, publication year, field, study design, and factors related to rehabilitation motivation. The study population was limited to stroke patients, the study site was not limited, and the purpose of the study was to explore and summarize the factors that influence the motivation for rehabilitation.

The dataset was structured through methodological categorization: research designs were classified as qualitative or quantitative approaches. Qualitative research was further divided into program effectiveness analyses, surveys, and panel surveys. Factors related to the motivation for rehabilitation were divided into seven sections: psychological, social relationships, illness and physiology, rehabilitation interventions, environment and behavior, demographic and economic, and other specific behaviors, with subcategories of risk factors and protective factors. All records were documented in Excel and archived in shared digital folders. To ensure data integrity, supplementary data requests were formally addressed to corresponding authors via institutional email protocols when encountering ambiguous or incomplete entries.

### 2.5 Collating, summarizing and reporting the results

#### 2.5.1 Collating

Data collection was performed using Excel (2016), with Microsoft Folders used for secure document sharing among research team members. All selected literature was carefully read more than twice to identify factors that influence motivation to recover. The analysis of collected data was conducted in two sequential phases: initial scoping review synthesis followed by systematic categorization of influencing factors.

##### 2.5.1.1 Phase 1: scoping review implementation

The scoping review methodology was employed to comprehensively map and analyze rehabilitation motivation factors in patients with stroke. A standardized evidence matrix was created using the following fields:

- Author(s) (Publication Year)
- Article Title
- Source Journal/Publication
- Study Design Type
- Identified Influencing Factors

This structured tabulation enabled systematic evidence mapping and facilitated cross-referencing.

#### 2.5.2 Summarizing

##### 2.5.2.1 Phase 2: factors integration and categorization

This phase involved the thematic synthesis of rehabilitation motivation factors across studies, as follows.

Frequency analysis of factor occurrence with source documentation.

Semantic consolidation of conceptually equivalent terms using:

Term unification (e.g., “depression” and “depressive symptoms” → depressive states).

Conceptual grouping (e.g., “financial pressure” and “economic status” → economic considerations).

The comprehensive categorization outcomes are detailed in Tables 1, 2, which present both the quantitative frequency distributions and qualitative conceptual integrations.

**Table 1:** Information on the literature included in the scoping review.

Article number	References	Article	Chinese title	Source	Document type	Factors related to rehabilitation motivation
1	Oh, SY et al. (23)	A prediction model of rehabilitation motivation in middle-aged survivors of stroke in rehabilitation facilities in Korea	韩国康复机构中年脑卒中幸存者康复动机的预测模型	Journal of Cardiovascular Nursing	Article	Self-efficacy, social support Depressive symptoms, social functioning
2	Cheong, MJ et al. (12)	Psychosocial factors related to stroke patients’ rehabilitation motivation: a scoping review and meta-analysis focused on South Korea	影响脑卒中患者康复动机的心理社会因素:韩国的范围回顾和Meta分析	Healthcare	Review	Depression, cognition, self-efficacy, self-esteem, disability acceptance, willpower, communication skills, adaptability, sense of empowerment, uncertainty. Sleep patterns, quality of life, ability to perform activities of daily living (ADLs), physical functioning, social support, financial burden, disease-related characteristics, rehabilitation environment.
3	Zheng, ZX et al. (32)	An investigation of the level of stigma and the factors influencing it in the rehabilitation of young and middle-aged stroke patients-a cross-sectional study.	老年脑卒中康复动机现状及其影响因素分析:横断面研究	BMC Neurology	Article	Social support, exercise compliance, kinesiophobia
4	Kang, M-S and Jung, HR (17)	The relationship of quality of sleep with stress and rehabilitation motivation in stroke	配偶婚姻亲密感直接影响住院中风幸存者的康复动机	Korean Journal of Occupational Therapy	Research-article	Marital intimacy, spousal depressive symptoms
5	Cheong, MJ et al. (13)	A protocol for systematic review and meta-analysis on psychosocial factors related to rehabilitation motivation of stroke patients.	脑卒中患者康复动机相关心理社会因素的系统评价和荟萃分析方案	Medicine	Review	Self-efficacy, social support, psychological distress, rehabilitation adherence
6	Przewoźnik, DA et al. (22)	The influence of cognitive, emotional and social factors on motivation for rehabilitation in patients after stroke	认知、情绪和社会因素对脑卒中患者康复动机的影响	Neuropsychiatria i Neuropsychologia	Review	Executive dysfunction, Depressive symptoms, Social support, Perceptions of illness and recovery
7	Lee, D-Y et al. (18)	Related factors of the motivation for rehabilitation in stroke patients	脑卒中患者康复动机的相关因素	Physical Therapy Korea	Research-article	Economic Stress, Depression, Couple Relationships
8	Otaka, Y et al. (33)	Patient and professional views of motivation for rehabilitation of subacute stroke	患者和专业人员对亚急性脑卒中康复动机的看法	Annals of Physical and Rehabilitation Medicine	Qualitative study	Rehabilitation goals, doctor/patient relationship, patient/friend relationship, support system, success/failure experiences, physical/cognitive status, patient resilience
9	Seo, M-Y et al. (34)	Factors influencing rehabilitation motivation of veterans after a stroke	影响中风后退伍军人康复动机的因素	The Journal of Fundamentals of Nursing	Research-article	Self-efficacy, social support, depression; monthly income and time of diagnosis
10	Yoshida, T et al. (28)	Motivation for rehabilitation in patients with subacute stroke: a qualitative study	亚急性脑卒中患者的康复动机: 定性研究	Frontiers in Rehabilitation Sciences	Journal Article	Patient Goals, Successful Failure Experiences, Physical Condition and Cognitive Function, Resilience. Rehabilitation Professional Influence, Interpatient Relationships, Patient Supporters.
11	Segura, E et al. (26)	The presence of anhedonia in individuals with subacute and chronic stroke: an exploratory cohort study	亚急性和慢性脑卒中患者中存在快感缺失:一项探索性队列研究	Frontiers in Aging Neuroscience	Article	Anhedonia
12	Kang, M-S and Jung, HR (17)	The relationship of quality of sleep with stress and rehabilitation motivation in stroke	脑卒中患者睡眠质量与应激及康复动机的关系	Korean Journal of Occupational Therapy	research-article	Quality of Sleep, Stress
13	Ntsinde, XC et al. (21)	Patient motivation in therapy and its impact on rehabilitation outcomes post-stroke: the therapists’ perspective	卒中后患者的治疗动机及其对康复结果的影响:治疗师的观点	University of the Witwatersrand	Dissertation/Thesis	Patient attitudes (voluntariness, compliance, participation), family support, social environment, therapist attitudes, cognitive functioning, patient acceptance of disease
14	Oyake, K et al. (27)	A multicenter explanatory survey of patients’ and clinicians’ perceptions of motivational factors in rehabilitation	患者与临床医生对康复动机因素认知的多中心解释性调查	Communications Medicine	Article	Recovery Realization, Goal Setting, Life-Related Practices, Information Transparency, Task Difficulty Management
15	Chen, HM et al. (35)	Effectiveness of motivational interviewing in regard to activities of daily living and motivation for rehabilitation among stroke patients	动机访谈对脑卒中患者日常生活活动能力和康复动机的影响	International Journal of Environmental Research and Public Health	Article	Motivational interviewing significantly improves. Self-efficacy, depressive symptoms. Social support (family support, medical team communication)
16	Oyake, K et al. (31)	Motivational strategies for stroke rehabilitation: a Delphi study	中风康复的激励策略:德尔菲研究	Archives of Physical Medicine and Rehabilitation	Article	Individual factors: cognitive functioning, personality traits, physical functioning Environmental factors: family environment, social environment Disease-related: type of diagnosis, comorbidities Treatment-related: task difficulty gradient, feedback mechanisms, goal-oriented exercises.
17	Last, N et al. (36)	Exploring patient perspectives of barriers and facilitators to participating in hospital-based stroke rehabilitation	探讨患者对参与以医院为基础的中风康复的障碍和促进者的看法	Disability and Rehabilitation	Article	Return to life roles
18	Alahmari, WS et al. (24)	Experiences and perceptions of post-stroke fatigue among stroke survivors in Saudi Arabia a qualitative interview study	沙特阿拉伯卒中幸存者对卒中后疲劳的体验和认知:一项定性访谈研究	Journal of Multidisciplinary Healthcare	Article	Post-stroke fatigue
19	Zheng, ZX et al. (32)	An investigation of the level of stigma and the factors influencing it in the rehabilitation of young and middle-aged stroke patients-a cross-sectional study	中青年脑卒中患者康复过程中耻感水平及其影响因素的横断面研究	BMC Neurology	Article	Stigma
20	Kwon, HK et al. (37)	The effect of a movie-based nursing intervention program on rehabilitation motivation and depression in stroke patients	基于电影的护理干预计划对脑卒中患者康复动机和抑郁的影响	Journal of Korean Academy of Nursing	Article	Movie intervention to improve motivation; depression, educational level, emotional cognition
21	Huang, XJ et al. (38)	Research progress on rehabilitation motivation for stroke patient	脑卒中患者康复动机的研究进展	Journal of Nursing Science	Review	Age, education, income; disease duration, lesion location; depression, family support; technology use, frequency of interventions. VR, play therapy, motivational interviewing are effective interventions.
22	Wang, H et al. (19)	Progress of research on assessment tools and factors influencing rehabilitation motivation of stroke patients	脑卒中患者康复动机评估工具及影响因素的研究进展	Today Nurse	Journal Article	Age, sex, economic status, education. Neurophysiology. Disease severity, comorbidities, communication disorders. Depression, apathy, self-efficacy. Family support, doctor-patient relationship, etc., rehabilitation environment.
23	Zhou, P et al. (39)	Advances in strategies to promote motivation for rehabilitation in stroke rehabilitation	促进康复动机的策略在卒中康复中的应用进展	Chinese Journal of Rehabilitation Medicine	Journal Article	Age, gender, personality, educational and social background, experience, coping skills, health status and lifestyle. Acceptance and adaptability to illness, lifestyle, experience, fatigue level, physical functioning. Clinical, family environment, rehabilitation environment, social support physician and therapist behavior.

**Table 2:** Integration and categorization of factors influencing motivation for rehabilitation.

Summary of influencing factors	No. of relevant literature
1. Psychological factors	
Depressive states (depression/depressive symptoms)	1, 2, 4, 5, 6, 7, 9, 10, 12, 15, 20
Self-efficacy	1, 3, 5, 6, 10, 12, 16, 17, 19
Motivation-related cognitions (willpower, empowerment, adaptability)	2, 8, 10
Resilience	8, 10
Anxiety/stress	12
Anhedonia	11
2. Social determinants	
Social support (family/friend/peer)	1, 3, 6, 9, 12, 13, 16, 17, 19
Interpersonal relationships (marital/couple/doctor-patient)	4, 7, 8, 10, 17
Social environment (community/resources)	2, 13, 16, 17
3. Economic considerations	
Financial status (burden/pressure/income)	2, 7, 9, 21, 23
Employment factors	17
4. Clinical characteristics	
Cognitive status (executive function/awareness)	2, 6, 8, 10, 16, 20, 22
Physical functioning (ADLs/mobility)	2, 8, 10, 16, 17
Disease severity (duration/comorbidities)	2, 16, 21, 22, 23
Neurobiological factors (lesion location/brain activity)	21, 22
5. Treatment-related factors	
Therapeutic alliance (therapist attitudes/communication)	13, 15, 16
Intervention characteristics (task difficulty/feedback)	14, 16
Technology integration (VR/play therapy)	21
Rehabilitation adherence	5
6. Demographic variables	
Age	21, 22, 23
Gender	22, 23
Education	21, 23
7. Systemic factors	
Healthcare system (service accessibility)	2, 14, 17
8. Rehabilitation environment	
Goal-setting processes	8, 14, 16
Success experiences	8, 10
Facility characteristics	2, 17

#### 2.5.3 Reporting the results

Through an extensive one-month literature search and reading, 45 influencing rehabilitation motivation were ultimately identified. In order to reduce repetition and redundancy and to facilitate reading, we have carried out conceptual unification, i.e., we have merged the factors that have the same central meaning but are expressed differently. Following multiple rounds of deliberation, we decided to categorize the influencing factors into eight categories: 1. Psychological Factors; 2. Social Determinants; 3. Economic Considerations; 4. Clinical Characteristics; 5. Treatment-related Factors; 6. Demographic Variables; 7. Systemic Factors and 8. Rehabilitation Environment. The specific influencing factors included in each category are shown in Table 2. The following section will present the results through four analytical dimensions: (1) frequency distribution of identified factors, (2) directional analysis of the effects, (3) actional pathways of influence, and (4) group heterogeneity.

##### 2.5.3.1 Frequency distribution of identified factors

After synthesizing 23 papers, the study revealed that among various factors associated with rehabilitation motivation in stroke patients, the three most frequently reported elements were depression, self-efficacy, social support, and cognitive status. The predominant factor was depressive state (including depression and depressive symptoms), documented in 11 studies. This was followed by self-efficacy and social support (from family/friends/peers), both of which were mentioned in nine studies and jointly ranked second. Cognitive status (particularly executive function and consciousness) emerged as the third most reported factor in eight studies. Subsequent analysis identified factors that occurred five times: physical functioning (activities of daily living and mobility), interpersonal relationships (marital/couple/doctor-patient dynamics), financial status (burden/pressure/income), and disease severity (duration/comorbidities). Conversely, anxiety/stress, anhedonia, employment factors, and rehabilitation adherence have only been documented in a single study. The remaining factors demonstrated a lower prevalence (2–3 occurrences), with the exception of the social environment (community/resources), which appeared in four studies.

##### 2.5.3.2 Directional analysis of the effects

###### 2.5.3.2.1 Facilitative impact

Social support and exercise adherence demonstrated positive correlations with rehabilitation motivation (*r* = 0.619, *p* < 0.01 and *r* = 0.569, *p* < 0.01, respectively). There is also a significant positive correlation between marital intimacy, sleep quality and motivation to recover (*p* < 0.01) (17). Furthermore, through literature analysis, this study identified several protective factors exerting positive influences on rehabilitation motivation, including family support, resilience, desire to resume life roles, disease acceptance, therapist rapport, educational attainment, rehabilitation environment, intervention frequency, information transparency, goal setting, task difficulty modulation, and positive, extroverted personality traits (12, 18).

###### 2.5.3.2.2 Inhibitory effect

Negative emotional manifestations such as depression, apathy, fear, and illness stigma contributed to diminished rehabilitation motivation in stroke patients. Kinesiophobia, financial burden, and spousal depressive symptoms demonstrated significant negative correlations with post-stroke rehabilitation motivation (*r* = −0.677, *p* < 0.01) (11), serving as clinical risk factors for recovery. Sleep disturbances were found to exacerbate daytime somnolence and anxiety, exerting detrimental effects on motivational levels (17). Furthermore, disease severity, comorbidities, disease duration, lesion location, post-stroke fatigue, previous unsuccessful rehabilitation experiences, poor social environment and communication barriers were identified as additional risk factors adversely affecting rehabilitation motivation in varying degrees (19–21).

###### 2.5.3.2.3 Bidirectional regulation

Social support may exhibit a dose-effect relationship, where moderate levels demonstrate the strongest association, while insufficient or excessive support could adversely affect rehabilitation motivation levels (22).

##### 2.5.3.3 Actional pathways of influence

Study showed that patients’ poststroke self-efficacy (*β* = 0.500, *p* < 0.001) and social support provided by family and medical staff (*β* = 0.284, *p* < 0.010) directly influenced their motivation to recover (23). Depressive symptoms, on the other hand, indirectly influenced motivation to recover through self-efficacy, as did physical functioning (18). Apathy significantly reduces goal-directed behaviors across the cognitive, emotional, and social domains, thereby exerting direct detrimental effects on rehabilitation motivation. Furthermore, in the model presented in the study by Oh, Soo Yong, Activities of Daily Living (ADL) did not exert a direct influence on rehabilitation motivation (17). Post-stroke fatigue and spousal depressive symptoms demonstrate indirect influences on motivational outcomes (24). Notably, research confirms that while patients’ depressive symptoms show no significant association with their own rehabilitation engagement motivation, spousal depressive symptoms directly impair marital intimacy, which subsequently mediates substantial negative impacts on patients’ rehabilitation motivation (20, 25). Similarly, clinical observations indicate that post-stroke anhedonia demonstrates significant comorbidity with depressive symptomatology. This neuropsychiatric interplay manifests as blunted reward sensitivity towards routine stimuli, in turn reduces intrinsic motivation to participate in rehabilitation programs and maintain a healthy active lifestyle (26).

##### 2.5.3.4 Group heterogeneity

Significant population heterogeneity in rehabilitation motivation exists among stroke patient groups. For instance, compared with elderly patients, young and middle-aged patients demonstrated higher rehabilitation motivation. Additionally, those under 65 years old prioritize “goal setting” and “achieving recovery” more than their older counterparts (27). In comparison to middle-aged patients, some elderly individuals may not exhibit their high levels of motivation through facial expressions or verbal communication. Consequently, the motivation of elderly patients may not always be readily observable. Relying solely on observational evaluation could result in the misinterpretation of their motivational levels (27, 28). A further instance could be married stroke patients with intimate spousal relationships exhibit significantly greater rehabilitation motivation than unmarried patients. Female stroke patients, often constrained by caregiving roles, tend to neglect their own healthcare needs, face prolonged stroke-related impacts, and display poorer rehabilitation initiative compared to males (29, 30). Moreover, patients with shorter hospital stays are more concerned about the availability of “medical information.” (27)

## 3 Discussion

The aim of this study was to summarize the influencing factors affecting the motivation for rehabilitation of stroke patients; therefore, a scope review was conducted to analyze the literature and papers of related studies at home and abroad to systematically and comprehensively identify the influencing factors of the motivation for rehabilitation of stroke patients.

First, in this scoping review, we systematically identified and included 23 relevant papers through rigorous methodological screening. The selected literature included 18 articles in English, three articles in Chinese, and 2 articles in Korean. By research design, the corpus included four qualitative studies, 12 quantitative studies, 5 review articles (including systematic reviews and meta-analyses), and 2 other research designs. Temporal analysis showed that 17 articles (73.9%) were published between 2020 and 2023, indicating academic interest in the topic during this period. In terms of study populations, seven articles specifically investigated older stroke patients and five studies targeted young and middle-aged stroke survivors. This distribution highlights both a primary research interest in older populations and a new focus on motivation for rehabilitation in younger stroke populations.

Second, a total of 44 influencing factors were identified in this study, of which psychological factors accounted for 10, and social factors and clinical characteristics accounted for 8 each. The combined evidence emphasizes that psychosocial factors (e.g., self-efficacy, depression) have been consistently identified as key determinants of motivation to recover in different populations of stroke patients. It is noteworthy that 86.9% of the included studies (20/23) in this study were published after 2020, which in part reflects the increased academic interest in this area. While a majority of studies focused on elderly stroke patients (7/23), emerging evidence (5/23) underscores the unique challenges faced by young and middle-aged survivors, such as stigma and socioeconomic burdens. This demographic divergence suggests the need for age-specific rehabilitation strategies. The included literature exhibited methodological diversity, encompassing qualitative explorations (e.g., patient perspectives), quantitative cross-sectional surveys, and systematic reviews. However, only two experimental studies (e.g., motivational interviewing, movie-based interventions) directly tested interventions, indicating a paucity of high-level evidence for clinical translation. In addition, regional differences between different regions were evident: the Korean study primarily emphasized family dynamics, whereas the Western study prioritized patient-physician communication. This cultural difference may affect the generalizability of the findings globally.

These variables have significant effects on motivation for rehabilitation. The literature has identified a number of gaps in current research, critical gaps persist in understanding the role of neurobiological mechanisms (e.g., anhedonia, post-stroke fatigue) and technology-driven interventions (e.g., VR, AI). Only one study (26) examined anhedonia, despite its potential link to motivational deficits. The predominance of cross-sectional designs (12/23) limits causal inference. Future longitudinal studies are needed to track motivation dynamics throughout rehabilitation, alongside randomized trials to validate scalable interventions (e.g., play therapy, telehealth).

## 4 Limitation

Most studies focus on short-term outcomes, lacking long-term tracking of dynamic changes in motivation. Although some research targets middle-aged and young patients, in-depth analyses of cultural and gender differences remain insufficient. In addition, grey literature (e.g., theses) was underrepresented, potentially omitting novel perspectives. In addition, according to the scope review framework, the literature was not evaluated for quality, which can reduce the validity of the findings. Most importantly, the research design and research methodology of qualitative or quantitative studies conducted by each researcher varies greatly, and the scoping review is only a descriptive summary, there is no strict requirement to evaluate the quality of the literature (14). Future studies should develop AI-driven personalized motivation, assessment models and explore interdisciplinary strategies (e.g., family-community-hospital collaboration).

## 5 Conclusion

The rehabilitation motivation of stroke patients is influenced by multidimensional factors. Key findings are summarized as follows:

Intrinsic Drivers: Self-efficacy and psychological status are core intrinsic determinants. Patients with higher self-efficacy exhibit greater initiative in participating in rehabilitation training, whereas negative emotions (e.g., anxiety, depression) significantly undermine motivation, though these can be ameliorated through psychological interventions.
External Environmental Support: Social support (including familial, community, and healthcare team engagement) serves as a critical external motivator. Active family involvement and emotional reinforcement enhance patient adherence, while economic conditions and accessibility to social resources (e.g., community-based rehabilitation facilities) directly influence long-term rehabilitation commitment.
Disease Characteristics: Disease severity and functional deficit types profoundly impact motivation. Patients with rapid recovery of motor function tend to sustain higher motivation levels, whereas complex symptoms such as executive dysfunction may lead to motivation decline due to frustration.
Intervention Strategies: Evidence-based approaches, including cognitive-behavioral therapy (CBT), motivational interviewing, gamified interventions, and integrated rehabilitation programs (combining physical, occupational, and psychological therapies), have demonstrated efficacy in enhancing motivation (31).
